# Dopamine D2 receptor on CD4^+^ T cells is protective against inflammatory responses and signs in a mouse model of rheumatoid arthritis

**DOI:** 10.1186/s13075-023-03071-1

**Published:** 2023-05-26

**Authors:** Xiao-Qin Wang, Huan-Huan Cai, Qiao-Wen Deng, Ya-Zhou Chang, Yu-Ping Peng, Yi-Hua Qiu

**Affiliations:** 1 Department of Physiology, School of Medicine, Nantong University, 19 Qixiu Road, Nantong, 226001 China; 2 School of Medicine, Southeast University, Nanjing, 210009 China

**Keywords:** Dopamine D2 receptor, Rheumatoid arthritis, Collagen-induced arthritis, CD4^+^ T cells, *D2r*^*fl/fl*^*/CD4*^*Cre*^ mice

## Abstract

**Background:**

Dopamine is a neurotransmitter and has been found to regulate lymphocytes by acting on dopamine receptors (DRs). CD4^+^ T cells express all the five subtypes of DRs, D1R to D5R. Although CD4^+^ T cells have been involved in pathogenesis of rheumatoid arthritis (RA), roles of DRs expressed on these cells in RA are poorly understood. This study determined whether D2R expressed on CD4^+^ T cells regulates inflammatory responses and signs in collagen type II (CII)-induced arthritis (CIA), a mouse model of RA.

**Methods:**

DBA/1 mice and C57BL/6 mice with global *D1r* or *D2r* deficiency (*D1r*^–/–^ or *D2r*^*–/–*^) or CD4^+^ T cell-specific *D2r* deletion (*D2r*^*fl/fl*^*/CD4*^*Cre*^) were used to prepare CIA model by intradermal injection of CII. D2R agonist sumanirole was intraperitoneally administered in CIA mice. CD4^+^ T cells obtained from CIA mice were exposed to sumanirole or/and D2R antagonist L-741,626 in vitro. Arthritic symptoms were assessed by clinical arthritis scores. Flow cytometric assay measured frequencies of CD4^+^ T cell subsets (Th1, Th2, Th17 and Treg cells). Expression of specific transcription factors for the CD4^+^ T cell subsets was tested by Western blot. Cytokine production was estimated by quantitative PCR and ELISA.

**Results:**

CIA mice manifested a bias of CD4^+^ T cells towards Th1 and Th17 cells. *D2r*^*–/–*^ CIA mice showed a stronger bias towards Th1 and Th17 phenotypes than CIA mice, while* D1r*^*–/–*^ CIA mice did not show the changes. CD4^+^ T cell-specific *D2r* deletion exacerbated both the polarization towards Th1 and Th17 cells and the symptoms of arthritis. Sumanirole administration in CIA mice ameliorated the bias of CD4^+^ T cells towards Th1 and Th17 phenotypes as well as arthritic symptoms. Sumanirole treatment of in vitro CD4^+^ T cells obtained from CIA mice promoted the shift to Treg cells, and the effect of sumanirole was blocked by L-741,626.

**Conclusions:**

D2R expressed on CD4^+^ T cells is protective against imbalance between pro-inflammatory and anti-inflammatory T cells and arthritic symptoms in CIA.

## Introduction

Rheumatoid arthritis (RA), a chronic autoimmune disease, is characterized by joint inflammation, synovial infiltration of immune cells, and cartilage and bone destruction [1]. Collagen type II (CII)-induced arthritis (CIA) is a murine model of RA and is widely used in research of human RA, as it shares many pathological and symptomatic similarities with human RA [2, 3]. Although the exact pathogenesis of RA is unknown, a pivotal role of CD4^+^ T cells in induction or perpetuation of this chronic autoimmune disease has been suggested [4, 5]. CD4^+^ T cells, on activation and expansion, develop into different T cell subsets, including helper T (Th)1, Th2, Th17, and regulatory T (Treg) cells, with different cytokine profiles and distinct effector functions. Th1 and Th17 cells exert pro-inflammatory properties, and Th2 and Treg cells are anti-inflammatory [6]. Early studies show that balance between Th1 and Th2 subsets is impaired in RA and Th1 driven inflammation is implicated in immunopathogenesis of RA [7–9]. Subsequent studies indicate that RA patients manifest an increase in peripheral Th17 frequencies and Th17-related cytokine levels while a decrease in Treg frequencies and Treg-related cytokine levels, suggesting that development of RA is associated with peripheral Th17/Treg imbalance [10, 11].

Dopamine is well recognized as a neurotransmitter in the central nervous system. Over five decades, this neurotransmitter has been also found in various systems in the periphery [12]. Growing evidence demonstrates that dopamine exists in the immune system and regulates a variety of immune functions [12–18]. Dopamine receptors (DRs), a significant G protein-coupled receptor, are essential for dopamine regulation of cell function. Based on DR-downstream cAMP formation in cells, DRs are classified into the two families: D1-like receptors (including D1R and D5R) and D2-like receptors (including D2R, D3R and D4R) [12]. T cells express the five DR subtypes [19–22]. Importantly, human CD4^+^ T cells always express all the five DRs and activation of the T cells increases the expression of the DRs [23]. Our previous work has showed that dopamine is synthesized and released by lymphocytes and modulates lymphocyte function by acting on DRs in an autocrine/paracrine way [24–27].

Different DR subtypes expressed on different immune cells are associated with the progression or recovery of RA or CIA. For example, D1R antagonist suppresses the severity of CIA [28]; dopamine released by dendritic cells causes aggravation of synovial inflammation of RA [29]; D2R expression level on B lymphocytes negatively correlates with RA activity [30]; D3R-positive mast cells in the synovial fluid show a declining trend with the increased disease activity in RA patients [31]; and D3R expressed on mast cells alleviates inflammation in CIA [32]. Our previous study showed that activation of D2-like receptors mitigated CIA symptoms by ameliorating Th17/Treg imbalance [33]. Nevertheless, further investigations are needed to clarify the role of D2R expressed on CD4^+^ T cells in RA or CIA. In this study, we demonstrate that D2R expressed on CD4^+^ T cells is protective against the imbalance between pro-inflammatory and anti-inflammatory T cells and the symptoms of arthritis in CIA mice by means of the approaches of global *D1r* or *D2r* deficiency (*D1r*^*–/–*^ or *D2r*^*–/–*^), CD4^+^ T cell-specific *D2r* deletion (*D2r*^*fl/fl*^*/CD4*^*Cre*^), D2R agonist administration, and in vitro treatments of CD4^+^ T cells with D2R agonist or/and antagonist. The current study provides a novel insight into the role of dopaminergic signaling of T cells in induction or prevention of RA or CIA.

## Materials and methods

### Mice

Male DBA/1 mice were obtained from the Center of Experimental Animals, Nantong University, China. Both the heterozygous *D1r* and *D2r* mice on C57BL/6 genetic background were kindly provided by Professor J.W. Zhou (Chinese Academy of Sciences, Shanghai, China). *D1r*^*−/−*^and *D2r*^*−/−*^ mice were obtained by cross-breeding of the heterozygous *D1r* and *D2r* mice, respectively. *D2r*-floxed (*D2r*^*fl/fl*^) mice were gifts from Professor J.W. Zhou (Chinese Academy of Sciences, Shanghai, China). *CD4*^*Cre*^ transgenic mice were gifts from Professor Z.C. Hua (Nanjing University, Nanjing, China). For T cell-specific deletion of *D2r*, the homozygous floxed *D2r* mice were crossed with *CD4*^*Cre*^ mice to obtain *D2r*^*fl/fl*^*/CD4*^*Cre*^ mice. Genotypes of all the mice were identified by polymerase chain reaction (PCR) amplification, as described previously [34]. The mice were housed with ad libitum access to food and water at 23 °C under a 12 h-light and 12 h-dark cycle. Male mice between 8 and 10 weeks of age were used in all experiments.

### Induction of CIA

For DBA/1 mice, CIA was induced as described in our previous publications [35, 36]. Briefly, mice were intradermally injected in the tail base with 0.1 ml emulsion containing 100 μg CII (Sigma-Aldrich, USA) in complete Freund's adjuvant (Chondrex, USA) on day 0 as a primary immunization. The mice were intraperitoneally boosted with 100 μg CII in incomplete Freund's adjuvant (Chondrex, USA) on day 21. Eventually, an intraperitoneal injection with 20 μg lipopolysaccharide dissolved in 20 μl PBS was executed to the mice on day 28. Mice were sacrificed on the 41st day after the primary immunization for subsequent experiments.

For C57BL/6 mice with *D1r*^*−/−*^, *D2r*^*−/−*^, or *D2r*^*fl/fl*^*/CD4*^*Cre*^ mutation, CIA was induced with a little difference from that for DBA/1 mice, based on [37]. Similarly, mice were intradermally injected in the tail base with 0.1 ml emulsion containing 100 μg CII in complete Freund's adjuvant on day 0 as a primary immunization. Dissimilarly, the mice received intraperitoneal injection with 60 μg CII in incomplete Freund's adjuvant and two hind-footpad injections with 40 μg CII on day 21. Mice of wild-type (WT) littermates served as control to induce CIA. Likewise, mice were sacrificed on the 41st day after the primary immunization for subsequent experiments.

### Evaluation of CIA symptoms

Arthritic symptoms were assessed by clinical arthritis score, hind paw thickness, and ankle joint width. The clinical arthritis score was measured by two investigators blinded to different groups every two days beginning from day 21 until day 41 after primary immunization using the following system for each limb: grade 0, no swelling; grade 1, slight swelling and erythema; grade 2, pronounced swelling; and grade 3, joint rigidity. A sum of clinical arthritis scores of four limbs for each mouse was submitted to statistical analysis. Mouse hind paw thickness and ankle joint width were also measured by the investigators using a microcalliper on the 41st day after the primary immunization.

### Treatment of CIA mice

The selective D2R agonist sumanirole (Sigma-Aldrich, USA), which was dissolved in saline, was intraperitoneally administered in DBA/1 CIA mice (1.5 mg/kg weight or 3 mg/kg weight) every other day with twice a day beginning from day 23 until day 41 after primary immunization. Saline injection in the same way was used as control.

### CD4^+^ T cell purification, activation, and treatments

The spleens of DBA/1 CIA mice were dissected and CD4^+^ T cells were obtained by magnetic cell sorting using a CD4^+^ T cell enrichment kit (BD Biosciences, USA) according to the manufacturer's protocol. Sorted cells were suspended in RPMI 1640 medium containing 10% heat-inactivated calf serum at a final concentration of 5 × 10^6^ cells/ml and stimulated with anti-CD3 and anti-CD28 antibodies (both 2 μg/ml; both from BD Pharmingen, USA) at 37 °C for 48 h to activate the cells. Simultaneously, the CD4^+^ T cells were exposed to the D2R agonist sumanirole (Sigma-Aldrich, USA) with the concentrations of 10^–7^ M or 10^–6^ M or to the D2R antagonist L-741,626 (Tocris Bioscience, USA) with the concentration of 10^–6^ M, which was incubated at 37 °C for 48 h. A combined treatment with L-741,626 (10^–6^ M, 30 min earlier) and sumanirole (10^–6^ M) was also performed in the cultured CD4^+^ T cells to show a blockage of the D2R agonist by the D2R antagonist.

### Flow cytometric assay for CD4^+^ T cell subsets and CD4^+^D2R^+^ cells

This method referred to our previous work [38]. The spleens were isolated from mice and mononuclear cells were prepared. The splenic mononuclear cells were incubated in round-bottom plates (2 × 10^5^ cells/well) with 50 ng/ml phorbol 12-myristate 13-acetate (Sigma-Aldrich, USA), 1 μM ionomycin (Sigma-Aldrich, USA), and 2 μM monensin (BD Biosciences, USA) for 5 h. After surface staining with APC-conjugated anti-CD4 antibody or/and FITC-conjugated anti-CD25 antibody (eBioscience, USA), the cells were fixed and permeabilized with the Cytofix/Cytoperm kit (BD Biosciences, USA). The cells were then stained intracellularly with PE-conjugated antibodies specific for interferon (IFN)-γ, interleukin (IL)-4, IL-17, or Foxp3 (all from eBioscience, USA). After PBS washes, the samples were run on a FACSCalibur flow cytometer equipped with an argon laser and analyzed by CellQuest software (BD Biosciences, USA).

For CD4^+^D2R^+^ cell measurement, splenic mononuclear cells were stained with APC-labeled anti-CD4 antibody (eBioscience, USA) and FITC-labeled anti-D2R antibody (Invitrogen, USA) for 30 min on ice. After PBS washes, the cells were analyzed for CD4^+^D2R^+^ cell percentage in total CD4^+^ cells using a FACSCalibur flow cytometer with CellQuest software (BD Biosciences, USA).

### Western blot analysis

The ankle joints were pulverized and homogenized in lysis buffer [33]. The supernatants were collected by centrifuging at 4 °C at 12,000 × g for 15 min to obtain protein extracts. The cultured CD4^+^ T cells were harvested to extract total protein with the lysis buffer. The protein samples (30 μg per lane) were fractionated by SDS-PAGE on 12% gels and then transferred onto polyvinylidene difluoride membranes (Millipore, USA). The membranes were blocked with 5% skim milk and separately incubated with the primary rabbit antibodies specific for D1R (1:200; Millipore, USA), D2R (1:200; Millipore, USA), T-bet (1:200; Santa Cruz Biotechnology, USA), GATA-3 (1:200; Santa Cruz Biotechnology, USA), ROR-γt (1:200; Invitrogen, USA), or Foxp3 (1:200; Santa Cruz Biotechnology, USA), or with the primary mouse antibody specific for β-actin (1:5000; Sigma-Aldrich, USA) overnight at 4 °C. Then, the membranes were incubated with IRDye 800- or 680-conjugated secondary antibodies (1:5000; Rockland Immunochemicals, USA) for 1 h at room temperature, followed by visualization using Odyssey laser scanning system (LI-COR Inc., USA). The relative quantities of the protein bands were determined by an image analysis system (Odyssey 3.0 software). Each target protein level was normalized to a ratio to β-actin.

### Quantitative real-time PCR

Total RNA was isolated from cultured CD4^+^ T cells with Trizol reagent (Invitrogen, USA) and reverse-transcribed using 5 × Prime Script RT Master Mix (Takara, Japan), as recommended by the manufacturer. The specific primers used to amplify the genes are as follows: IFN-γ, 5’-TCAAGTGGCATAGATGTGGAAGAA-3’ and 5’-TGGCTCTGCAGGATTTTCATG-3’ (NM_008337.2); IL-4, 5’-ACAGGAGAAGGGACGCCAT-3’ and 5’-GAAGCCCTACAGACGAGCTCA-3’ (NM_021283.1); IL-17A, 5’-GCTCCAGAAGGCCCTCAGA’ and 5’-AGCTTTCCCTCCGCATTGA-3’ (NM_010552.3); transform growth factor (TGF)-β1, 5’-GATACGCCTGAGTGGCTGTC-3’ and 5’-GCTGATCCCGTTGATTTCC-3’ (NM_011577.2); β-actin, 5’-CTGTCCCTGTATGCCTCTG-3’ and 5’-ATGTCACGCACGATTTCC-3’ (NM_007393.5). The PCR was performed in triplicate by using the Rotor-Gene 3000 Real-time Cycler with SYBR green I as the detection system. β-actin was used as an endogenous standard.

### Enzyme-linked immunosorbent assay (ELISA)

The ankle joints were pulverized and homogenized in lysis buffer. The supernatants were collected by centrifuging at 4 °C at 12,000 × g for 15 min to measure contents of the cytokines IFN-γ, IL-4, IL-17A and TGF-β1 in the joints. The serum was also collected to test anti-CII IgG level. The samples were assayed using commercially available ELISA kits (eBioscience, USA), following the manufacturer's instructions.

### Histopathology

This procedure referred to our previous work [35]. Briefly, ankle joints of mice were removed, fixed in 4% phosphate-buffered formalin for 3 days, and decalcified in 10% EDTA for 15 days. Sections with 20 µm in thickness were prepared in a freezing microtome (Leica CM 1900–1-1, Germany) and stained with H&E.

### Statistical analysis

Data were expressed as mean ± standard deviation. Statistical analysis was performed using GraphPad Prism software. Comparisons between two groups were evaluated using Student's t test. Multiple comparisons among the groups were assessed by one-way analysis of variance, followed by a post hoc analysis. Significance was assigned at *P* < 0.05.

## Results

### Effects of global *D1r* or *D2r* ablation on differentiation and function of CD4^+^ T cells in CIA mice

**Fig. 1:**
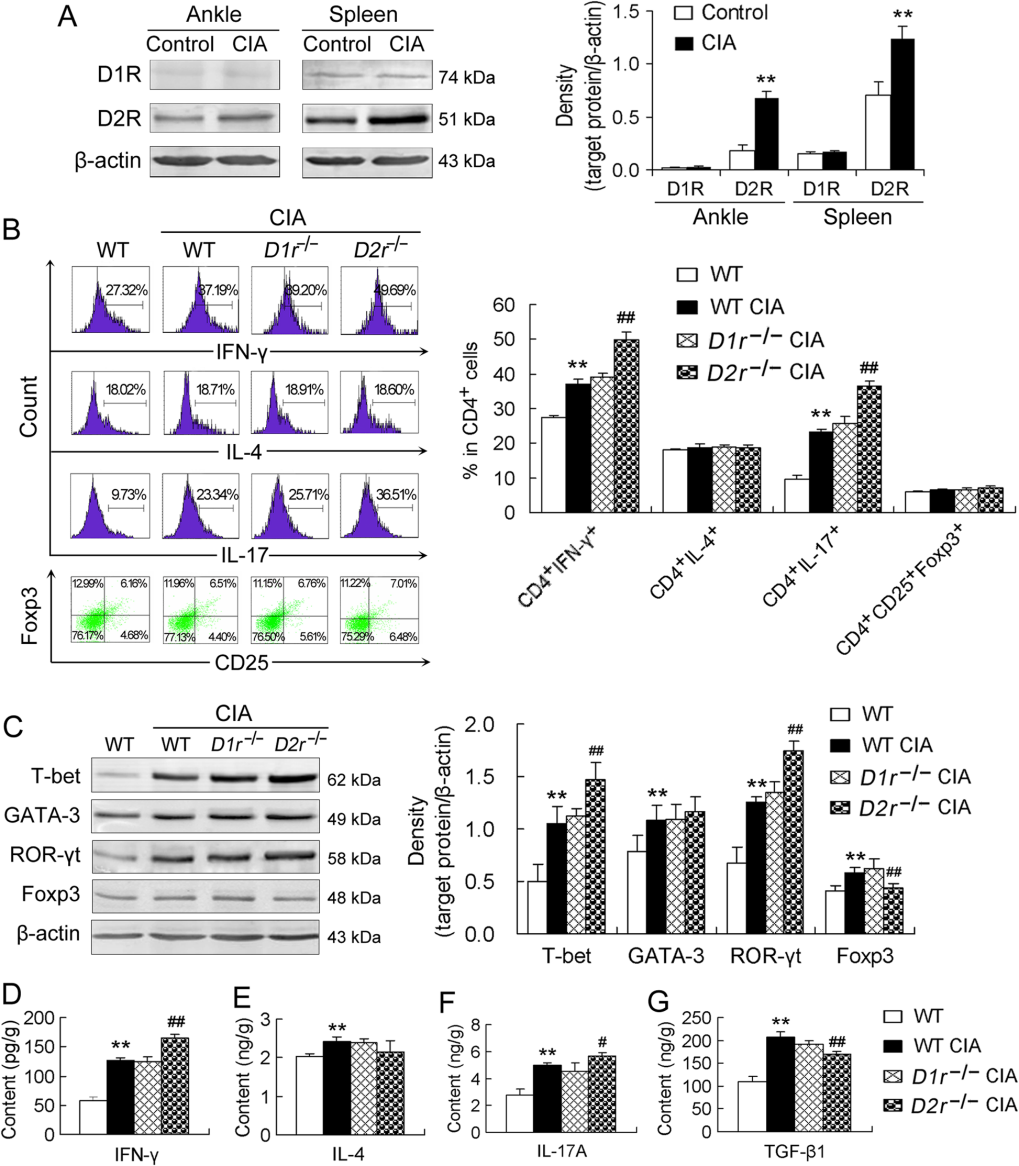
Effects of global *D1r* or *D2r* ablation on differentiation and function of CD4^+^ T cells in CIA mice. **A** Protein expression of D1R and D2R in ankle joints and spleen of mice. The left panel indicates Western blotting bands and the right panel is densitometric data normalized to β-actin (*n* = 5/group). **B** Flow cytometric assay of the CD4^+^ T cell subsets, CD4^+^IFN-γ^+^ (Th1) cells, CD4^+^IL-4^+^ (Th2) cells, CD4^+^IL-17^+^ (Th17) cells, and CD4^+^CD25^+^Foxp3^+^ (Treg) cells. Representative images of the flow cytometric assay are exhibited in the left panel and quantitative data showing percentages of the CD4^+^ T cell subsets in total CD4^+^ T cells are indicated in the right panel (*n* = 5/group). **C** Protein expression of the specific transcription factors T-bet, GATA-3, ROR-γt and Foxp3 for Th1, Th2, Th17 and Treg cells, respectively, in ankle joints. Representative Western blotting bands are exhibited in the left panel and densitometric data normalized to β-actin are indicated in the right panel (*n* = 5/group). **D-G** Contents of the cytokines IFN-γ, IL-4, IL-17A and TGF-β1 in ankle joints measured by ELISA. *n* = 6/group. **P < 0.01, versus control or WT mice; ^#^*P* < 0.05, ^##^*P* < 0.01, versus WT CIA mice

D2R expression was upregulated in both ankle joints and spleen of CIA mice compared with that of control mice, while D1R expression was not significantly different in both the tissues between CIA mice and control mice (Fig. 1A). Differentiation of CD4^+^ T cells towards the four subsets, Th1, Th2, Th17 and Treg cells, was determined by percentages of CD4^+^IFN-γ^+^ (Th1) cells, CD4^+^IL-4^+^ (Th2) cells, CD4^+^IL-17^+^ (Th17) cells and CD4^+^CD25^+^Foxp3^+^ (Treg) cells in total CD4^+^ T cells as well as by expression of T-bet, GATA-3, ROR-γt and Foxp3, the specific transcription factors for Th1, Th2, Th17 and Treg cells, respectively. The percentages of CD4^+^IFN-γ^+^ cells and CD4^+^IL-17^+^ cells, the two pro-inflammatory CD4^+^ T cell subsets, were increased in CIA mice compared with those in control mice (Fig. 1B). Importantly, global *D2r* ablation augmented the CIA-induced increases in Th1 and Th17 cell frequencies, while global* D1r* loss did not significantly alter the CIA-induced changes (Fig. 1B). In contrast, the percentages of CD4^+^IL-4^+^ cells and CD4^+^CD25^+^Foxp3^+^ cells, the two anti-inflammatory CD4^+^ T cell subsets, were not significantly altered by any condition of CIA, global *D2r* deficiency or global* D1r* deficiency (Fig. 1B). In addition, protein expression of T-bet and ROR-γt in ankle joints was upregulated in CIA mice relative to that in control mice (Fig. 1C). Global *D2r* deficiency further upregulated the T-bet and ROR-γt expression in CIA mice, but global* D1r* deficiency did not alter the CIA-induced T-bet and ROR-γt expression (Fig. 1C). Interestingly, protein expression of both GATA-3 and Foxp3 was upregulated in CIA mice compared with that in control mice, but only the upregulated Foxp3 expression in CIA mice was reduced by global *D2r* deletion (Fig. 1C).

Functional bias of CD4^+^ T cells towards the four subsets was determined by levels of IFN-γ, IL-4, IL-17A and TGF-β1 in the ankle joints, since the cytokines are produced mainly by Th1, Th2, Th17 and Treg cells, respectively. Similar to the changes in T-bet and ROR-γt expression, the levels of the two pro-inflammatory cytokines IFN-γ and IL-17A in the ankle joints were increased in CIA mice relative to those in control mice, and *D2r* deficiency further increased IFN-γ and IL-17A levels, but *D1r* deficiency did not further alter IFN-γ and IL-17A levels in CIA mice (Fig. 1D and F). Similar to the changes in GATA-3 and Foxp3 expression, the levels of the two anti-inflammatory cytokines IL-4 and TGF-β1 in the ankle joints were higher in CIA mice than in control mice, but only TGF-β1 level was significantly reduced by *D2r* deficiency (Fig. 1E and G).

### D2R agonist sumanirole ameliorates depolarization of Th1 and Th17 cells and symptoms of arthritis in CIA mice

**Fig. 2:**
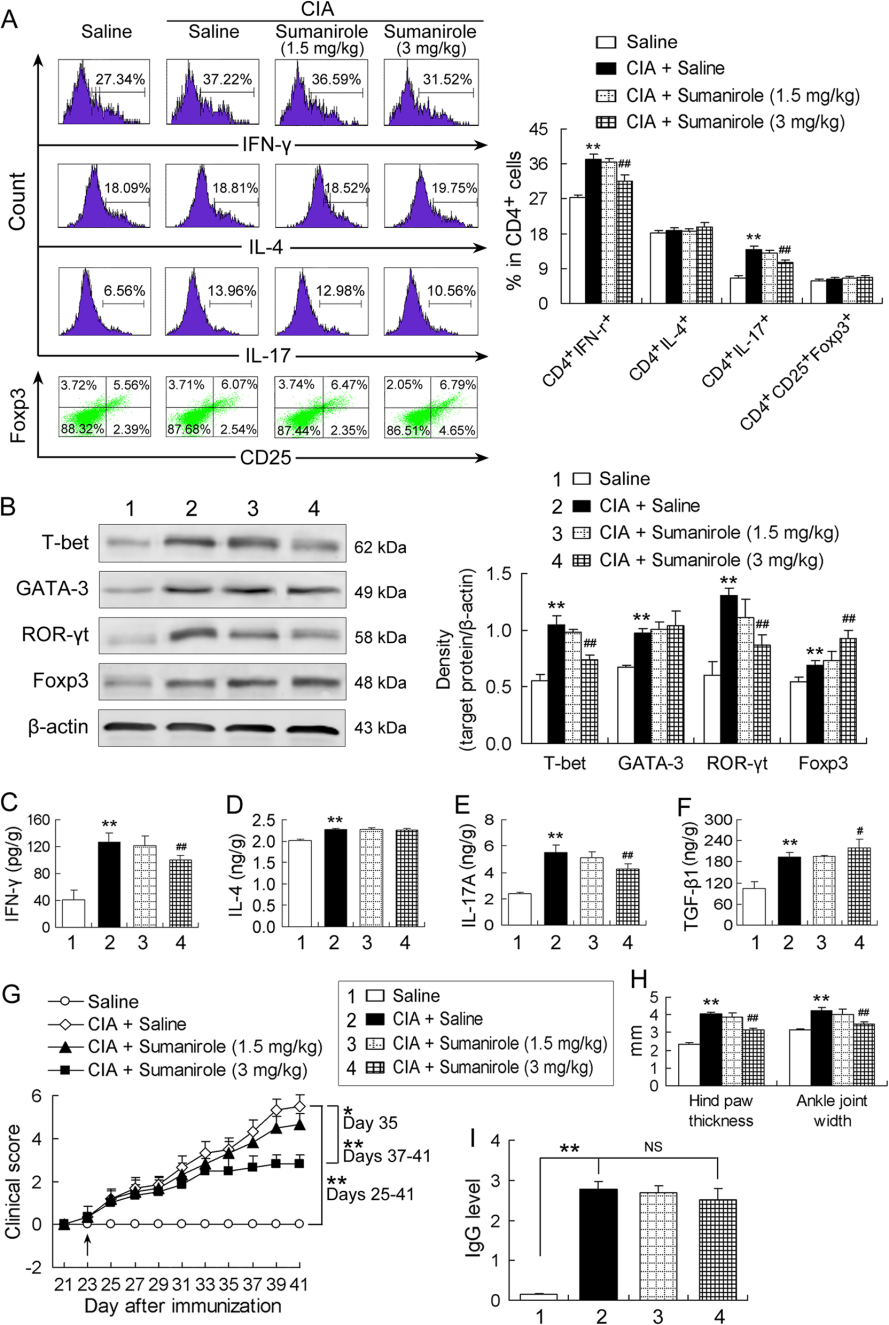
D2R agonist sumanirole ameliorates depolarization of Th1 and Th17 cells and symptoms of arthritis in CIA mice. **A** Flow cytometric assay of the CD4^+^ T cell subsets. The left panel represents the flow cytometric images and the right panel indicates respective percentage of the CD4^+^ T cell subsets (Th1, Th2, Th17 and Treg cells) in total CD4^+^ T cells (*n* = 5/group). **B** Protein expression of the specific transcription factors in ankle joints of mice. Representative Western blotting bands are exhibited in the left panel and quantitative values obtained by densitometric analysis were normalized to β-actin in the right panel (*n* = 5/group). **C-F** Contents of the cytokines IFN-γ, IL-4, IL-17A and TGF-β1 in ankle joints of mice measured by ELISA. All *n* = 5. **G** Clinical arthritis score of four limbs. The arrow indicates the first sumanirole administration. *n* = 6/group. **H** Hind paw thickness and ankle joint width of mice. *n* = 6/group. **I** Anti-CII IgG antibody levels in serum of mice measured by ELISA. *n* = 5/group. *P < 0.05, **P < 0.01, versus saline group; ^#^*P* < 0.05, ^##^*P* < 0.01, versus CIA + saline group; NS, no significance

Mice were intraperitoneally injected with the D2R agonist sumanirole (1.5 or 3 mg/kg weight) every other day with twice a day beginning from day 23 until day 41 after primary immunization. Saline injection in the same way served as control of the sumanirole treatment. Similarly, differentiation of CD4^+^ T cells towards Th1, Th2, Th17 and Treg cells was assessed by frequencies of CD4^+^IFN-γ^+^ cells, CD4^+^IL-4^+^ cells, CD4^+^IL-17^+^ cells and CD4^+^CD25^+^Foxp3^+^ cells as well as by expression of T-bet, GATA-3, ROR-γt and Foxp3. The increased frequencies of CD4^+^IFN-γ^+^ cells and CD4^+^IL-17^+^ cells in CIA mice were reduced by the treatment with the D2R agonist sumanirole of 3 mg/kg (but not 1.5 mg/kg) (Fig. 2A). However, the frequencies of CD4^+^IL-4^+^ cells and CD4^+^CD25^+^Foxp3^+^ cells were altered neither by CIA nor CIA plus sumanirole (Fig. 2A). Furthermore, the upregulated expression of T-bet and ROR-γt in CIA mice was also reduced by sumanirole of 3 mg/kg (but not 1.5 mg/kg) (Fig. 2B). In contrast, the upregulated Foxp3 expression in CIA mice was further increased by sumanirole, although the upregulated GATA-3 expression in CIA mice was not altered by sumanirole (Fig. 2B).

The changes in levels of the cytokines IFN-γ, IL-4, IL-17A and TGF-β1, which represent functions of Th1, Th2, Th17 and Treg cells, respectively, were similar to those in expression of their specific transcription factors in CIA or sumanirole administration. The raised levels of IFN-γ and IL-17A, the two pro-inflammatory cytokines, in the ankle joints of CIA mice were attenuated by sumanirole administration (Fig. 2C and E). Although the levels of IL-4 and TGF-β1, the two anti-inflammatory cytokines, were also raised by CIA, the sumanirole administration further increased TGF-β1 level (Fig. 2D and F).

In addition, symptoms of arthritis were estimated by clinical arthritis score, which was measured every other day from day 21 to day 41 after primary immunization, as well as by hind paw thickness and ankle joint width on day 41 post-immunization. The clinical arthritis score was continuously elevated in CIA mice from day 25 to day 41 after immunization in comparison with that in control mice (Fig. 2G). Sumanirole treatment with 3 mg/kg (but not 1.5 mg/kg) reduced the elevation of the clinical arthritis score during days 35 through 41 after immunization (Fig. 2G). Moreover, the increased hind paw thickness and ankle joint width in CIA mice were also reduced by sumanirole administration with 3 mg/kg (Fig. 2H). Furthermore, CIA mice showed a dramatic increase in anti-CII IgG antibody level in serum on day 41 post-immunization compared with control mice (Fig. 2I). However, the raised serum anti-CII IgG level in CIA mice was not significantly reduced by the sumanirole administration (Fig. 2I).

### CD4^+^ T cell-specific ***D2r*** ablation aggravates depolarization of Th1 and Th17 cells and symptoms of arthritis in CIA mice

**Fig. 3:**
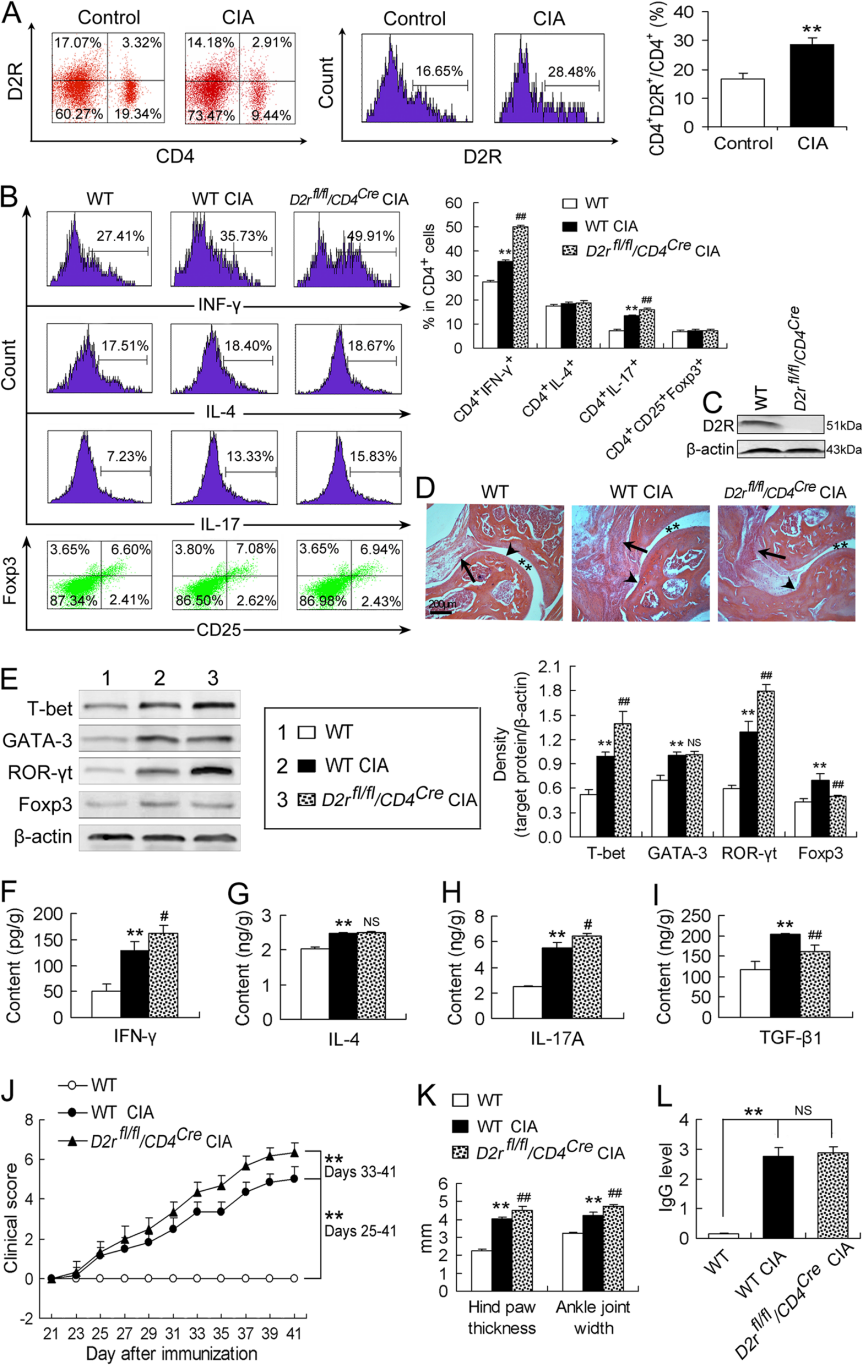
CD4^+^ T cell-specific *D2r* ablation aggravates depolarization of Th1 and Th17 cells and symptoms of arthritis in CIA mice. **A** Flow cytometric assay for CD4^+^D2R^+^ cells. The left two panels indicate representative images of the flow cytometric assay, and the right panel is quantitative data showing percentage of CD4^+^D2R^+^ cells in total CD4^+^ cells (*n* = 6/group). **B** Flow cytometric assay of the CD4^+^ T cell subsets. The left panel indicates representative images of the flow cytometric assay, and the right panel is quantitative data showing respective percentage of Th1, Th2, Th17 and Treg cells in total CD4^+^ cells (*n* = 5/group). **C** Efficiency of CD4^+^ T cell-specific *D2r* deletion. CD4^+^ T cells that were obtained from the spleen of WT or *D2r*^*fl/fl*^*/CD4*^*Cre*^ mice by magnetic cell sorting were analyzed by Western blot. **D** Histopathology of ankle joints. The arrows point at synovial tissue showing synovial hyperplasia in CIA mice and *D2r*^*fl/fl*^*/CD4*^*Cre*^ CIA mice. The arrow heads denote articular cartilage or bone showing cartilage destruction in CIA mice and bone erosion in *D2r*^*fl/fl*^*/CD4*^*Cre*^ CIA mice. The asterisks indicate articular cavity. **E** Protein expression of the four transcription factors in ankle joints. Representative protein bands are indicated in the left panel and densitometric data normalized to β-actin are shown in the right panel (*n* = 5/group). **F-I** Contents of the cytokines in ankle joints determined by ELISA. All *n* = 5. **J** Clinical arthritis score of four limbs, **K** hind paw thickness and ankle joint width, and **L** serum anti-CII IgG levels determined by ELISA. *n* = 6 in (**J**), **K** and (**L**). **P < 0.01, versus control or WT mice; ^#^*P* < 0.05, ^##^*P* < 0.01, versus WT CIA mice; NS, no significance

The percentage of CD4^+^D2R^+^ cells in total CD4^+^ T cells was increased in CIA mice relative to that in control mice (Fig. 3A), suggesting an involvement of CD4^+^ T cell-expressed D2R in CIA. To clarify the role of D2R expressed on CD4^+^ T cells in CIA pathogenesis, we evaluated effects of CD4^+^ T cell-specific *D2r* ablation on T cell imbalance, joint histopathology and arthritic symptoms in CIA mice. It was found that the increased frequencies of CD4^+^IFN-γ^+^ (Th1) cells and CD4^+^IL-17^+^ (Th17) cells in CIA mice were further increased by CD4^+^ T cell-specific *D2r* deficiency (Fig. 3B). In contrast, the frequencies of CD4^+^IL-4^+^ (Th2) cells and CD4^+^CD25^+^Foxp3^+^ (Treg) cells were not significantly altered by any condition of CIA or CD4^+^ T cell-specific *D2r* deficiency (Fig. 3B). Western blotting analysis indicated that D2R expression was deficient in CD4^+^ T cells obtained from the conditional *D2r*-deficient mice (Fig. 3C), confirming the efficiency of CD4^+^ T cell-specific *D2r* deletion. Furthermore, the histopathology of ankle joints showed synovial hyperplasia and cartilage destruction in CIA mice as well as bone erosion in *D2r*^*fl/fl*^*/CD4*^*Cre*^ CIA mice (Fig. 3D). Moreover, the upregulated expression of T-bet and ROR-γt in the ankle joints of CIA mice was increased by CD4^+^ T cell-specific *D2r* deletion (Fig. 3E). However, the upregulated Foxp3 expression in CIA mice was attenuated by CD4^+^ T cell-specific *D2r* loss (Fig. 3E), although the upregulated GATA-3 expression in CIA mice was not altered by CD4^+^ T cell-specific *D2r* loss (Fig. 3E). Likewise, the raised IFN-γ and IL-17A levels by CIA were increased by CD4^+^ T cell-conditional *D2r* deficiency (Fig. 3F and H), but the raised TGF-β1 level by CIA was reduced by CD4^+^ T cell-conditional *D2r* deficiency (Fig. 3I) and the raised IL-4 level by CIA was not changed by the gene mutation (Fig. 3G).

In addition, *D2r*^*fl/fl*^*/CD4*^*Cre*^ CIA mice manifested further increased clinical arthritis score during days 33 through 41 after immunization with respect to WT CIA mice (Fig. 3J). And also *D2r*^*fl/fl*^*/CD4*^*Cre*^ CIA mice exhibited further increased hind paw thickness and ankle joint width in comparison with WT CIA mice (Fig. 3K). However, the elevated anti-CII IgG antibody level in the serum of CIA mice on day 41 post-immunization was not affected by CD4^+^ T cell-conditional *D2r* deficiency (Fig. 3L).

### D2R agonist sumanirole in vitro inhibits CIA-induced bias of CD4^+^ T cells towards Th1 and Th17 phenotypes and D2R antagonist L-741,626 blocks this effect

**Fig. 4:**
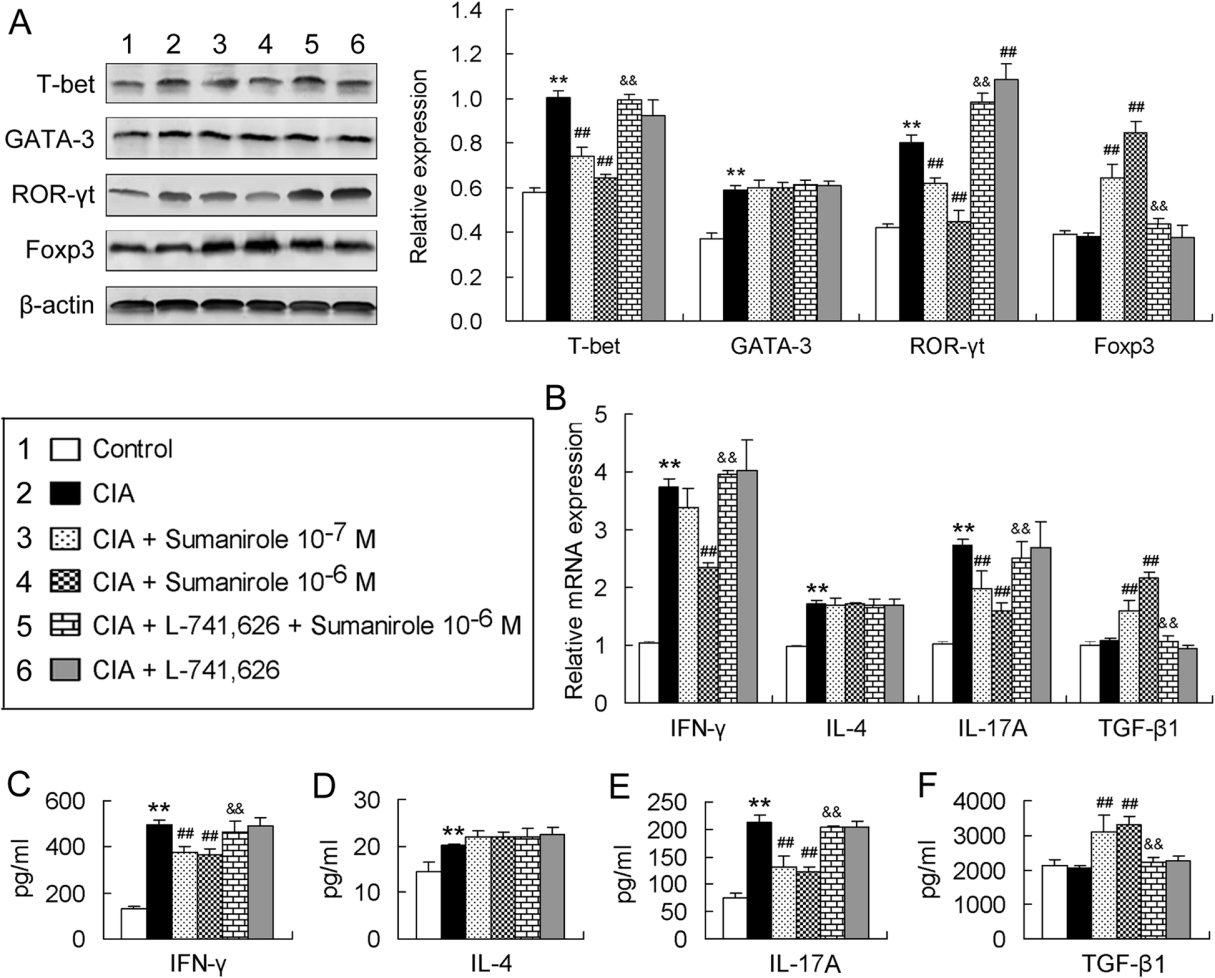
D2R agonist sumanirole in vitro inhibits CIA-induced bias of CD4^+^ T cells towards Th1 and Th17 phenotypes and D2R antagonist L-741,626 blocks this effect. **A** Protein expression of the specific transcription factors in CD4^+^ T cells. The left panel represents Western blotting bands and the right panel indicates quantitative data of the left panel. **B** Gene expression levels of the cytokines in CD4^+^ T cells. **C-F** Concentrations of the cytokines in supernatants of the activated CD4^+^ T cells. All the data were from five repeated experiments. **P < 0.01, versus control group; ^##^*P* < 0.01, versus CIA group; ^&&^*P* < 0.01, versus CIA + sumanirole 10^–6^ M group

The D2R agonist sumanirole (10^–7^ or 10^–6^ M) was applied alone or in combination with the D2R antagonist L-741,626 (10^–6^ M) to in vitro CD4^+^ T cells obtained from CIA mice in the presence of the stimulation with anti-CD3 and anti-CD28 antibodies. An alone L-741,626 exposure was also performed to determine whether this D2R antagonist itself affects CD4^+^ T cells. It was found that CD4^+^ T cells obtained from CIA mice expressed more T-bet and ROR-γt than those cells from control mice (Fig. 4A). The treatment with sumanirole of both the concentrations (10^–7^ and 10^–6^ M) inhibited the CIA-induced T-bet and ROR-γt expression in CD4^+^ T cells (Fig. 4A). The combined treatment of CIA-derived CD4^+^ T cells with L-741,626 and sumanirole returned to the high levels of T-bet and ROR-γt expression (Fig. 4A), showing that the D2R antagonist L-741,626 blocked the inhibitory effect of the D2R agonist sumanirole. Interestingly, alone L-741,626 exposure upregulated ROR-γt expression in CIA-derived CD4^+^ T cells in comparison with no L-741,626 exposure, but it did not significantly affect T-bet expression (Fig. 4A). Moreover, GATA-3 expression was also upregulated in CD4^+^ T cells in CIA condition, but any treatment with alone sumanirole, combined L-741,626 and sumanirole, or alone L-741,626 did not alter the CIA-induced expression of GATA-3 (Fig. 4A). Although CIA did not significantly affect Foxp3 expression in CD4^+^ T cells, sumanirole of both the concentrations obviously upregulated the Foxp3 expression, and this effect of sumanirole was abolished by L-741,626 (Fig. 4A).

Functional bias of in vitro CD4^+^ T cells towards Th1, Th2, Th17 and Treg phenotypes was assessed by gene expression and secretion levels of IFN-γ, IL-4, IL-17A and TGF-β1 in the T cells. The upregulated mRNA expression of both IFN-γ and IL-17A in CD4^+^ T cells in CIA condition was reduced by the D2R agonist sumanirole, and this effect of sumanirole was eliminated by the D2R antagonist L-741,626 (Fig. 4B). IL-4 mRNA expression was also upregulated in CD4^+^ T cells in CIA condition, but the upregulated IL-4 expression by CIA was not altered by any treatment with alone sumanirole, combined L-741,626 and sumanirole, or alone L-741,626 (Fig. 4B). In contrast, CIA did not significantly influence TGF-β1 mRNA expression in CD4^+^ T cells, but sumanirole upregulated TGF-β1 expression, and L-741,626 abrogated this effect of sumanirole (Fig. 4B). Furthermore, the changes in secretion levels of the four cytokines in CD4^+^ T cells were similar to those in the gene expression. CIA increased IFN-γ and IL-17A concentrations in supernatants of the activated CD4^+^ T cells, sumanirole reduced the CIA-induced secretion of IFN-γ and IL-17A from CD4^+^ T cells, and L-741,626 abolished these effects of sumanirole (Fig. 4C and E). The level of IL-4 secretion from CD4^+^ T cells was elevated in CIA condition, but any treatment with alone sumanirole, combined L-741,626 and sumanirole, or alone L-741,626 did not alter the elevated IL-4 level (Fig. 4D). In contrast, CIA did not affect TGF-β1 secretion from CD4^+^ T cells, but sumanirole increased the TGF-β1 secretion, and L-741,626 blocked the effect of sumanirole (Fig. 4F).

## Discussion

In the present study, global *D2r* deficiency exacerbated CIA-induced imbalance between pro-inflammatory and anti-inflammatory T cell responses, as evidenced by the increases in Th1 and Th17 cell frequencies, T-bet and ROR-γt expression, and IFN-γ and IL-17A levels, as well as by the decreases in Foxp3 expression and TGF-β1 production. In contrast, global *D1r* deficiency did not significantly alter the CIA-induced pro-inflammatory/anti-inflammatory imbalance. Moreover, D2R expression in both ankle joints and spleen was upregulated in CIA mice, while D1R expression in the two tissues was not altered by CIA. These findings suggest that D2R but not D1R is actively involved in the regulation of pro-inflammatory and anti-inflammatory T cell activities during CIA. In support of the present results, we previously showed that *D2r*-deficient CIA mice manifested more severe arthritic symptoms than WT CIA mice, while *D1r*-deficient CIA mice did not alter the arthritis [33]. It is clear that activation of D1-like receptors (D1R and D5R) increases the formation of their downstream signaling cAMP, whereas activation of D2-like receptors (D2R, D3R and D4R) inhibits the production of cAMP [12]. This may explain the different effects between *D1r* and *D2r* deficiency on CIA. Indeed, Nakano et al. [29] have indicated that D2-like receptor antagonist haloperidol significantly induces accumulation of IL-6^+^ and IL-17^+^ T cells with exacerbated cartilage destruction in RA synovial/SCID mouse chimera model, whereas D1-like receptor antagonist SCH-23390 strongly suppresses these responses. In addition, Wang et al. [32] recently have shown that *D3r*-deficient CIA mice develop more severe arthritis than WT CIA mice. The findings support our current results, since D2R and D3R share the same downstream cAMP signaling. However, the role of D1R in CIA or RA seems complicated. A recent report reveals that expression of D1R on B cells of RA patients correlates positively with disease duration and severity only in women, indicating sex-specific differences in dopaminergic pathway in RA, with a pro-inflammatory feature of the D1R pathway in women [39]. Thus, whether the male CIA mice used in this study are a reason for the unaffected effect by *D1r* deficiency is worthy of future investigations.

Contrary to the *D2r-*deficient effects, D2R agonist sumanirole, which was intraperitoneally injected in CIA mice, alleviated the imbalance of pro-inflammatory/anti-inflammatory T cells, as determined by the inhibition in Th1 and Th17 cell differentiation and function and the enhancement in Treg cell differentiation and function. As a result, the arthritic signs in CIA mice were mitigated by the sumanirole administration. These results demonstrate that D2R is involved in amelioration of both Th17/Treg imbalance and arthritic symptoms. On the other hand, the elevated serum anti-CII IgG level in CIA mice was not significantly reduced by the sumanirole administration. It implies that anti-CII IgG antibody is not easily neutralized by the D2R agonist. The speculation is supported by the findings from Nakashioya et al. [28]. They indicate that D1-like receptor antagonist SCH23390 does not affect serum levels of antibodies (IgG1, IgG2a and IgG2b) to CII, although it suppresses the severity of CIA.

Our further investigation showed that CD4^+^ T cell-specific *D2r* loss exacerbated not only the imbalance between pro-inflammatory and anti-inflammatory T cell responses but also the arthritic symptoms in CIA mice. These effects are consistent with those of global *D2r* ablation. This suggests that CD4^+^ T cell-expressed D2R dominates the regulation of CIA. Furthermore, in vitro data revealed that activation of D2R on CD4^+^ T cells obtained from CIA mice with the agonist sumanirole promoted the polarization towards Treg phenotype. The effect of sumanirole was blocked by D2R antagonist L-741,626. Accordingly, we propose that D2R expressed on CD4^+^ T cells is protective against inflammatory responses and signs in CIA. As a support, B cell-expressed D2R may be instrumental in improvement of RA signs [30].

CD4^+^ T cells have been crucially associated with the pathogenesis of RA or CIA and therefore, regulating CD4^+^ T cells is important to therapy of RA or CIA. Studies carried out on human and murine T cells have shown that activation of D2R increases production of IL-10 [14] and that DR2 can be effective in regulating the activation and differentiation of naive CD4^+^ T cells by promoting the polarization toward Treg cells [40]. Blocking D2R leads to high IL-17 production in the interface between dendritic cells and naive CD4^+^ T cells [29]. Because Th17 and Treg cells are involved in autoimmunity as autoaggressive and beneficial cells, respectively, it is likely that D2R is involved in the protection against autoimmunity [41]. Our current results provide further evidence for the protection against CIA progression by D2R expressed on CD4^+^ T cells. Based on the regulation, a potential therapeutic strategy for RA via activating D2R can be suggested.

## Conclusions

Global *D2r* deficiency exacerbated imbalance between pro-inflammatory and anti-inflammatory T cells in CIA mice, while global *D1r* deficiency did not alter the CIA-induced changes. Systemic administration of D2R agonist sumanirole in CIA mice alleviated pro-inflammatory/anti-inflammatory T cell imbalance and arthritic symptoms. CD4^+^ T cell-specific *D2r* deletion aggravated T cell inflammatory responses and arthritic symptoms in CIA mice, whereas the D2R agonist sumanirole in vitro promoted the shift of CIA CD4^+^ T cells to Treg phenotype. These findings demonstrate that D2R expressed on CD4^+^ T cells is protective against CIA progression. Therefore, activation of D2R signaling may become a potential therapeutic strategy for RA.

## Data Availability

The datasets used and/or analyzed during the current study are available from the corresponding author on reasonable request.

## Abbreviations

CII: Collagen type II
CIA: Collagen type II-induced arthritis
DRs: Dopamine receptors
D1R: Dopamine D1 receptor
D2R: Dopamine D2 receptor
*D1r*^*–*^^*/*^^*–*^: Global *D1r* deficiency
*D2r*^*–*^^*/*^^*–*^: Global* D2r* deficiency
*D2r*^*fl/fl*^: *D2r*-Floxed
*D2r*^*fl/fl*^*/CD4*^*C*^^*re*^: CD4^+^ T cell-specific *D2r* deletion
ELISA: Enzyme-linked immunosorbent assay
IFN-γ: Interferon-γ
IL-4: Interleukin-4
IL-17: Interleukin-17
PCR: Polymerase chain reaction
RA: Rheumatoid arthritis
TGF-β1: Transform growth factor -β1
Th1: Helper T1
Th2: Helper T2
Th17: Helper T17
Treg: Regulatory T
WT: Wild-type

## References

[1] Smolen JS, Aletaha D, McInnes IB (2016). Rheumatoid arthritis. Lancet.

[2] Cho YG, Cho ML, Min SY, Kim HY (2007). Type II collagen autoimmunity in a mouse model of human rheumatoid arthritis. Autoimmun Rev.

[3] Holmdahl R, Andersson ME, Goldschmidt TJ, Jansson L, Karlsson M, Malmström V, Mo J (1989). Collagen induced arthritis as an experimental model for rheumatoid arthritis. Immunogenetics, pathogenesis and autoimmunity. APMIS..

[4] Cope AP, Schulze-Koops H, Aringer M (2007). The central role of T cells in rheumatoid arthritis. Clin Exp Rheumatol.

[5] Chemin K, Gerstner C, Malmström V (2019). Effector Functions of CD4+ T Cells at the Site of Local Autoimmune Inflammation-Lessons from Rheumatoid Arthritis. Front Immunol.

[6] Noack M, Miossec P (2014). Th17 and regulatory T cell balance in autoimmune and inflammatory diseases. Autoimmun Rev.

[7] Schulze-Koops H, Kalden JR (2001). The balance of Th1/Th2 cytokines in rheumatoid arthritis. Best Pract Res Clin Rheumatol.

[8] Kusaba M, Honda J, Fukuda T, Oizumi K (1998). Analysis of type 1 and type 2 T cells in synovial fluid and peripheral blood of patients with rheumatoid arthritis. J Rheumatol.

[9] van der Graaff WL, Prins AP, Niers TM, Dijkmans BA, van Lier RA (1999). Quantitation of interferon gamma- and interleukin-4-producing T cells in synovial fluid and peripheral blood of arthritis patients. Rheumatology (Oxford).

[10] Wang W, Shao S, Jiao Z, Guo M, Xu H, Wang S (2012). The Th17/Treg imbalance and cytokine environment in peripheral blood of patients with rheumatoid arthritis. Rheumatol Int.

[11] Niu Q, Cai B, Huang ZC, Shi YY, Wang LL (2012). Disturbed Th17/Treg balance in patients with rheumatoid arthritis. Rheumatol Int.

[12] Matt SM, Gaskill PJ (2020). Where Is Dopamine and how do Immune Cells See it?: Dopamine-Mediated Immune Cell Function in Health and Disease. J Neuroimmune Pharmacol.

[13] Saha B, Mondal AC, Majumder J, Basu S, Dasgupta PS (2001). Physiological concentrations of dopamine inhibit the proliferation and cytotoxicity of human CD4+ and CD8+ T cells in vitro: a receptor-mediated mechanism. NeuroImmunoModulation.

[14] Besser MJ, Ganor Y, Levite M (2005). Dopamine by itself activates either D2, D3 or D1/D5 dopaminergic receptors in normal human T-cells and triggers the selective secretion of either IL-10 TNFalpha or both. J Neuroimmunol.

[15] Watanabe Y, Nakayama T, Nagakubo D, Hieshima K, Jin Z, Katou F, Hashimoto K, Yoshie O (2006). Dopamine selectively induces migration and homing of naive CD8+ T cells via dopamine receptor D3. J Immunol.

[16] Levite M (2016). Dopamine and T cells: dopamine receptors and potent effects on T cells, dopamine production in T cells, and abnormalities in the dopaminergic system in T cells in autoimmune, neurological and psychiatric diseases. Acta Physiol (Oxf).

[17] Pinoli M, Marino F, Cosentino M (2017). Dopaminergic Regulation of Innate Immunity: a Review. J Neuroimmune Pharmacol.

[18] Kawano M, Takagi R, Saika K, Matsui M, Matsushita S (2018). Dopamine regulates cytokine secretion during innate and adaptive immune responses. Int Immunol.

[19] Santambrogio L, Lipartiti M, Bruni A, Dal Toso R (1993). Dopamine receptors on human T- and B-lymphocytes. J Neuroimmunol.

[20] Levite M, Chowers Y, Ganor Y, Besser M, Hershkovits R, Cahalon L (2001). Dopamine interacts directly with its D3 and D2 receptors on normal human T cells, and activates beta1 integrin function. Eur J Immunol.

[21] McKenna F, McLaughlin PJ, Lewis BJ, Sibbring GC, Cummerson JA, Bowen-Jones D, Moots RJ (2002). Dopamine receptor expression on human T- and B-lymphocytes, monocytes, neutrophils, eosinophils and NK cells: a flow cytometric study. J Neuroimmunol.

[22] Kirillova GP, Hrutkay RJ, Shurin MR, Shurin GV, Tourkova IL, Vanyukov MM (2008). Dopamine receptors in human lymphocytes: radioligand binding and quantitative RT-PCR assays. J Neurosci Methods.

[23] Kustrimovic N, Rasini E, Legnaro M, Marino F, Cosentino M (2014). Expression of dopaminergic receptors on human CD4+ T lymphocytes: flow cytometric analysis of naive and memory subsets and relevance for the neuroimmunology of neurodegenerative disease. J Neuroimmune Pharmacol.

[24] Huang Y, Qiu AW, Peng YP, Liu Y, Huang HW, Qiu YH (2010). Roles of dopamine receptor subtypes in mediating modulation of T lymphocyte function. Neuro Endocrinol Lett.

[25] Liu Y, Huang Y, Wang XQ, Peng YP, Qiu YH (2012). Effect of tyrosine hydroxylase gene silencing in CD4+ T lymphocytes on differentiation and function of helper T cells. Neuro Endocrinol Lett.

[26] Huang HW, Tang JL, Han XH, Peng YP, Qiu YH (2013). Lymphocyte-derived catecholamines induce a shift of Th1/Th2 balance toward Th2 polarization. NeuroImmunoModulation.

[27] Zhao W, Huang Y, Liu Z, Cao BB, Peng YP, Qiu YH (2013). Dopamine receptors modulate cytotoxicity of natural killer cells via cAMP-PKA-CREB signaling pathway. PLoS One..

[28] Nakashioya H, Nakano K, Watanabe N, Miyasaka N, Matsushita S, Kohsaka H (2011). Therapeutic effect of D1-like dopamine receptor antagonist on collagen-induced arthritis of mice. Mod Rheumatol.

[29] Nakano K, Yamaoka K, Hanami K, Saito K, Sasaguri Y, Yanagihara N, Tanaka S, Katsuki I, Matsushita S, Tanaka Y (2011). Dopamine induces IL-6-dependent IL-17 production via D1-like receptor on CD4 naive T cells and D1-like receptor antagonist SCH-23390 inhibits cartilage destruction in a human rheumatoid arthritis/SCID mouse chimera model. J Immunol.

[30] Wei L, Zhang C, Chen HY, Zhang ZJ, Ji ZF, Yue T, Dai XM, Zhu Q, Ma LL, He DY, Jiang LD (2015). Dopamine receptor DR2 expression in B cells is negatively correlated with disease activity in rheumatoid arthritis patients. Immunobiology.

[31] Xue L, Li X, Chen Q, He J, Dong Y, Wang J, Shen S, Jia R, Zang QJ, Zhang T, Li M, Geng Y (2018). Associations between D3R expression in synovial mast cells and disease activity and oxidant status in patients with rheumatoid arthritis. Clin Rheumatol.

[32] Wang B, Li X, Li M, Geng Y, Wang N, Jin Y, Zhang W, Xu K, Wang J, Tao L, Lai S, Wu K, Lei J, Wang J, Zhou T, Li K, Chen Y, Xue L (2022). Dopamine D3 receptor signaling alleviates mouse rheumatoid arthritis by promoting Toll-like receptor 4 degradation in mast cells. Cell Death Dis.

[33] Lu JH, Liu YQ, Deng QW, Peng YP, Qiu YH (2015). Dopamine D2 Receptor Is Involved in Alleviation of Type II Collagen-Induced Arthritis in Mice. Biomed Res Int..

[34] Shao W, Zhang SZ, Tang M, Zhang XH, Zhou Z, Yin YQ, Zhou QB, Huang YY, Liu YJ, Wawrousek E, Chen T, Li SB, Xu M, Zhou JN, Hu G, Zhou JW (2013). Suppression of neuroinflammation by astrocytic dopamine D2 receptors via αB-crystallin. Nature.

[35] Zhao XY, Cui SW, Wang XQ, Peng YP, Qiu YH (2013). Tyrosine hydroxylase expression in CD4(+) T cells is associated with joint inflammatory alleviation in collagen type II-induced arthritis. Rheumatol Int.

[36] Wang XQ, Liu Y, Cai HH, Peng YP, Qiu YH (2016). Expression of tyrosine hydroxylase in CD4^+^ T cells contributes to alleviation of Th17/Treg imbalance in collagen-induced arthritis. Exp Biol Med (Maywood).

[37] Bevaart L, Vervoordeldonk MJ, Tak PP. Collagen-induced arthritis in mice. In: Proetzel G, Wiles MV, editors. Mouse Models for Drug Discovery, Methods in Molecular Biology. New York: Humana Press; 2010. p. 181–92. 10.1007/978-1-60761-058-8_11 20012399

[38] Liu Z, Zhai XR, Du ZS, Xu FF, Huang Y, Wang XQ, Qiu YH, Peng YP (2021). Dopamine receptor D2 on CD4^+^ T cells is protective against neuroinflammation and neurodegeneration in a mouse model of Parkinson's disease. Brain Behav Immun.

[39] Wieber K, Fleige L, Tsiami S, Reinders J, Braun J, Baraliakos X, Capellino S (2022). Dopamine receptor 1 expressing B cells exert a proinflammatory role in female patients with rheumatoid arthritis. Sci Rep.

[40] Jafari M, Ahangari G, Saberi M, Samangoui S, Torabi R, Zouali M (2013). Distorted expression of dopamine receptor genes in systemic lupus erythematosus. Immunobiology.

[41] Buttarelli FR, Fanciulli A, Pellicano C, Pontieri FE (2011). The dopaminergic system in peripheral blood lymphocytes: from physiology to pharmacology and potential applications to neuropsychiatric disorders. Curr Neuropharmacol.

